# Selenoproteins regulate macrophage invasiveness and extracellular matrix-related gene expression

**DOI:** 10.1186/1471-2172-10-57

**Published:** 2009-10-28

**Authors:** Bradley A Carlson, Min-Hyuk Yoo, Yasuyo Sano, Aniruddha Sengupta, Jin Young Kim, Robert Irons, Vadim N Gladyshev, Dolph L Hatfield, Jin Mo Park

**Affiliations:** 1 Molecular Biology of Selenium Section, Laboratory of Cancer Prevention, Center for Cancer Research, National Cancer Institute, National Institutes of Health, Bethesda, MD 20892, USA; 2 Cutaneous Biology Research Center, Massachusetts General Hospital and Harvard Medical School, Charlestown, MA 02129, USA; 3 Department of Biochemistry, University of Nebraska, Lincoln, NE 68588, USA

## Abstract

**Background:**

Selenium, a micronutrient whose deficiency in diet causes immune dysfunction and inflammatory disorders, is thought to exert its physiological effects mostly in the form of selenium-containing proteins (selenoproteins). Incorporation of selenium into the amino acid selenocysteine (Sec), and subsequently into selenoproteins is mediated by Sec tRNA^[Ser]Sec^.

**Results:**

To define macrophage-specific selenoprotein functions, we generated mice with the Sec tRNA^[Ser]Sec ^gene specifically deleted in myeloid cells. These mutant mice were devoid of the "selenoproteome" in macrophages, yet exhibited largely normal inflammatory responses. However, selenoprotein deficiency led to aberrant expression of extracellular matrix-related genes, and diminished migration of macrophages in a protein gel matrix.

**Conclusion:**

Selenium status may affect immune defense and tissue homeostasis through its effect on selenoprotein expression and the trafficking of tissue macrophages.

## Background

Macrophages, a class of myeloid leukocytes with phagocytic activity and inflammatory signaling properties, play a pivotal role in antimicrobial defense and tissue homeostasis [1-3]. These tissue-resident immune cells express receptors that detect the presence of signature molecules associated with microbial infection and tissue damage [4,5]. Such receptors signal to induce macrophage production of cytokines and other inflammatory mediators that effect pathogen clearance and tissue repair. Many cell-intrinsic and -extrinsic regulatory mechanisms act to limit inflammatory signaling in macrophages, thereby preventing excessive, self-destructive responses of the cell. Furthermore, the spatial and temporal turnover of macrophages in tissue is subject to dynamic control by the local microenvironment and metabolic state. Uncontrolled macrophage recruitment and activation is associated with development of rheumatic, cardiovascular, metabolic, and neoplastic disorders.

Selenium is a dietary trace element that exerts both beneficial and adverse effects on health depending on the specific chemical form and dose. Deficiency or excess of dietary selenium has been linked to immune dysfunction and inflammatory disorders [6,7] although the precise molecular mechanisms remain to be determined. In living organisms, selenium either exists as low-molecular weight compounds such as selenite, selenomethionine, methylselenol or selenomethylselenocysteine, or is assimilated into selenium-containing proteins (selenoproteins) by way of the amino acid selenocysteine (Sec). Apart from nonspecific selenium incorporation into protein, the pathway of Sec and selenoprotein biosynthesis requires a unique tRNA, Sec tRNA^[Ser]Sec ^[8,9].

Using a conditional gene knockout strategy, we have shown that ablation of Sec tRNA^[Ser]Sec ^results in the loss of expression of the whole selenoprotein set, or selenoproteome, in hepatocytes [10], and T cells [11]. Therefore, cell type-specific deletion of the Sec tRNA^[Ser]Sec ^gene (*Trsp*) offers a useful way to examine the physiological effects of selenium in the form of selenoproteins in relation to the function of a desired cell type. In this paper, we present data from a study of mice lacking Sec tRNA^[Ser]Sec ^in macrophages and describe the molecular and cellular abnormalities caused by the ablation of macrophage selenoprotein expression.

## Results

### Selenoprotein expression in macrophages

**Figure 1:**
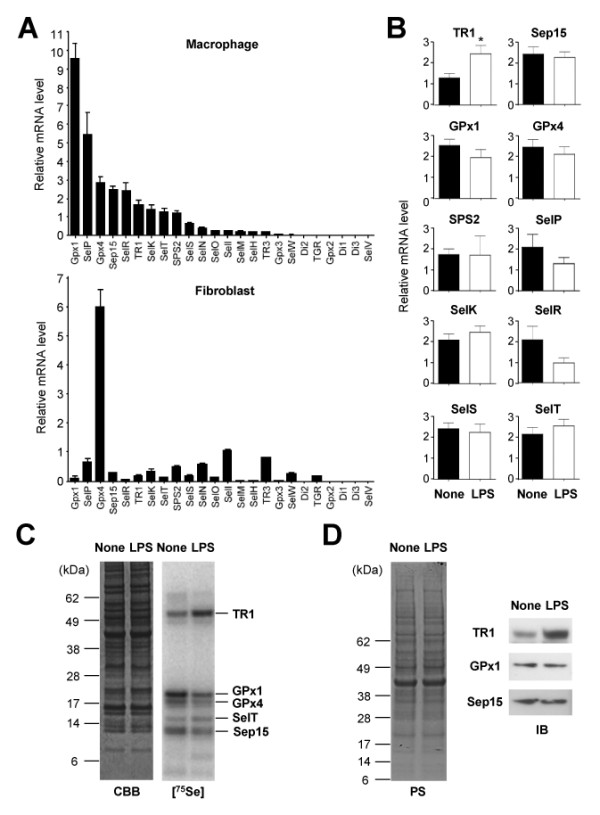
**Profiling of selenoprotein gene expression in macrophages**. (A) Selenoprotein gene expression in macrophages (BMDMs) and fibroblasts (NIH3T3 embryonic fibroblasts) was analyzed by real-time qPCR and is shown as relative mRNA level to GUSB (β-glucuronidase; internal control). (B) Macrophages left unstimulated (None) and stimulated with LPS (100 ng/ml; 4 h) were subjected to RNA extraction and real-time qPCR. Data represent mean ± standard deviation (*n *= 4). *, *p *< 0.05. (C) ^75^Se-labeled selenoproteins in macrophages are visualized by autoradiography after SDS electrophoresis. Macrophages were left unstimulated (None) and stimulated with LPS (100 ng/ml; 6 h) before protein extraction. The identities of the major, labeled selenoproteins are designated on the right of panel. CBB, Coomassie Brilliant Blue. (D) Whole cell lysates from macrophages treated with LPS as in (C) were analyzed by immunoblotting with antibodies against the proteins indicated on the left. PS, Ponceau S.

The mouse genome contains 24 selenoprotein genes [12]. Individual selenoproteins exhibit tissue specificity in their expression levels and regulation patterns [13]. To examine selenoprotein mRNA expression in primary mouse macrophages, we isolated total RNA from bone marrow-derived macrophages (BMDMs) and analyzed the expression of all 24 mouse selenoprotein genes by real-time qPCR (Figure 1A). Compared to 3T3 fibroblasts, a non-myeloid cell type that we used in parallel, macrophages expressed relatively high levels of genes encoding glutathione peroxidases 1 (GPx1), thioredoxin reductase 1 (TR1), the 15-kDa selenoprotein (Sep15), and selenoproteins P, R, K, and T (Figure 1A). To compare selenoprotein gene expression in resting and activated macrophages, BMDMs were subjected to RNA extraction with and without treatment with lipopolysaccharide (LPS), a toll-like receptor 4 (TLR4) agonist that induces potent inflammatory responses. TR1 gene expression was significantly increased in BMDMs after 4 h of incubation with LPS (Figure 1B).

Selenoprotein expression in macrophages was further examined by labeling BMDMs with ^75^Se, and analyzing labeled selenoproteins by gel electrophoresis [14]. BMDMs expressed a distinct set of selenoproteins, whose repertoire was largely unchanged by LPS activation (Figure 1C). However, the amount of TR1 was substantially increased, whereas that of GPx1 was modestly decreased in LPS-treated macrophages, which was verified by immunoblotting with antibodies specific to individual selenoproteins (Figure 1D). In addition to TR1 and GPx1, several other selenoproteins with high mRNA abundance in macrophages were detected as ^75^Se-labeled protein bands on the gel.

### Ablation of *Trsp *and selenoprotein expression in macrophages

To study the macrophage-specific functions of selenoproteins, we generated mutant mice in which deletion of floxed (*fl*) alleles of *Trsp *is driven by Cre recombinase expressed under the control of the lysozyme M (*LysM*) promoter. *LysMCre *expression is restricted to macrophages and other leukocyte subpopulations of myeloid origin [15].*Trsp*^*fl*/*fl*^-*LysMCre *(*ΔTrsp*^*M*^) mice were born at Mendelian ratios, survived to adulthood, and displayed no spontaneous pathology under the specific pathogen-free condition (data not shown).

**Figure 2:**
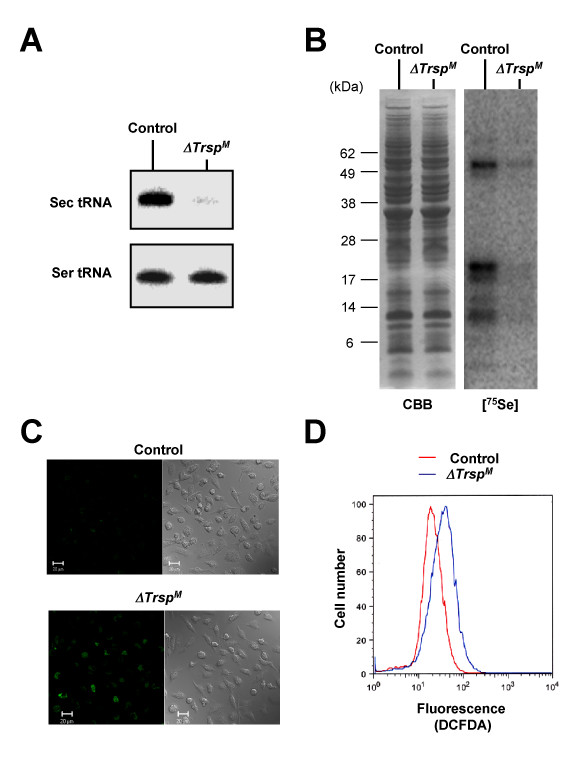
**Characterization of *ΔTrsp*^*M *^macrophages**. (A) The levels of tRNA^[Ser]Sec ^and tRNA^*Ser *^in macrophages (BMDMs) derived from control and *ΔTrsp*^*M *^mice as determined by Northern blotting are shown. (B) ^75^Se-labeled selenoproteins are visualized by autoradiography after SDS electrophoresis as in Figure 1B. CBB, Coomassie Brilliant Blue. (C) ROS production was analyzed by confocal microscopy following DCFDA staining. Fluorescence (left panels) and phase contrast (right panels) images are shown. (D) ROS production was analyzed by flow cytometry following DCFDA staining.

We examined the effects of *Trsp *deletion on selenoprotein expression and function in macrophages. In *ΔTrsp*^*M *^macrophages, synthesis of Sec tRNA^[Ser]Sec ^was almost completely abolished (Figure 2A). Correspondingly, the level of ^75^Se-labeled selenoproteins was dramatically diminished in *ΔTrsp*^*M *^macrophages as compared to that of control (*Trsp*^*fl*/*fl*^) cells (Figure 2B), indicating that the Sec tRNA^[Ser]Sec^-mediated cotranslational mechanism is the major pathway for selenium incorporation into protein in macrophages. The low amounts of residual ^75^Se-labeled proteins in *ΔTrsp*^*M *^macrophages (Figure 2B) likely represent selenoproteins derived from non-macrophage cells contaminating the BMDM preparation and also from a small fraction of cells that have escaped *Trsp *deletion. Many selenoproteins function to quench reactive oxygen species (ROS) and other forms of oxidants, or function to repair oxidative damage [12]. We previously found that selenoprotein-deficient T cells accumulated higher ROS than control cells and failed to mount ROS-sensitive T cell receptor responses [11]. Similarly, steady state levels of ROS were higher in resting *ΔTrsp*^*M *^macrophages than in control cells (Figure 2C and 2D). Therefore, the antioxidant function of selenoproteins is required to maintain redox homeostasis in various cell types including macrophages.

### Inflammatory responses in *ΔTrsp*^*M *^macrophages and mice

**Figure 3:**
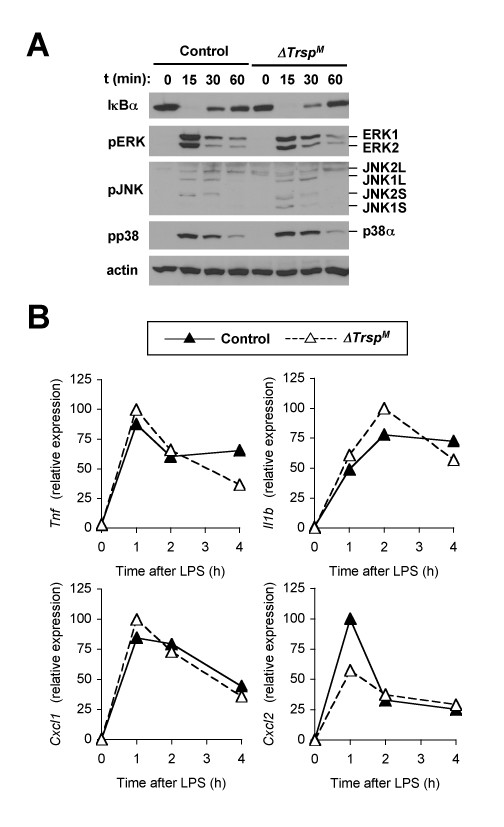
**Inflammatory signaling and inflammatory gene expression in selenoprotein-deficient macrophages**. (A) Whole cell lysates from BMDMs treated with LPS were prepared after the indicated durations of stimulation and analyzed by immunoblotting with antibodies against the proteins indicated on the left. Data are representative of five experiments. (B) BMDMs were treated with LPS (100 ng/ml) for the indicated durations, and gene expression was analyzed by qPCR. Data are representative of three experiments. There were no statistically significant differences in the genes tested.

Macrophages contribute to the initiation of inflammation by producing cytokines in response to microbial infection and tissue injury. LPS and other inflammatory stimuli bring about transient ROS accumulation as part of cellular signaling events [16,17]. Selenoproteins such as GPx isoforms were previously shown to regulate inflammatory responses [18,19]. We therefore explored the effect of selenoprotein deficiency and the resultant redox imbalance on the macrophage inflammatory response. Signaling to the transcription factor NF-κB, and the mitogen-activated protein kinase (ERK, JNK and p38) cascades are crucial for cellular responses to inflammatory stimuli. In LPS-treated macrophages, degradation and replenishment of IκBα, both indicative of NF-κB activation, occurred independently of selenoprotein status (Figure 3A). Induction of phosphorylated ERK, JNK and p38, the active forms of the protein kinases, by LPS was also normal in *ΔTrsp*^*M *^macrophages (Figure 3A). We also compared LPS-induced inflammatory gene expression in control and *ΔTrsp*^*M *^macrophages. The magnitude and kinetics of expression of the genes encoding the cytokines tumor necrosis factor-α and interleukin-1β, and the chemokines KC and macrophage inflammatory protein-2 (*Tnf*, *Il1b*, *Cxcl1*, and *Cxcl2*, respectively) were similar in both macrophage groups (Figure 3B).

**Figure 4:**
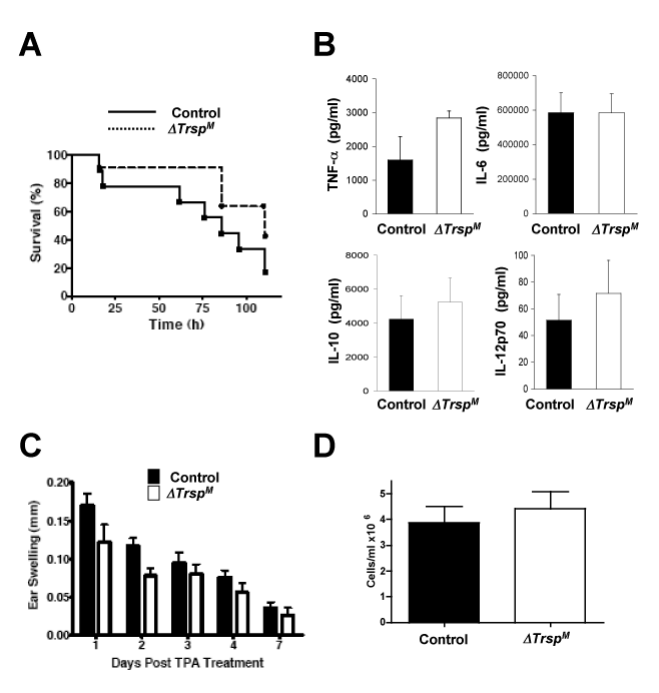
**Inflammatory responses in *ΔTrsp*^*M *^mice**. (A) LPS (50 mg/kg) was intraperitoneally injected into mice. Mortality of the animals was determined at the indicated time points (*n *= 9 for control mice; *n *= 11 for *ΔTrsp*^*M *^mice). (B) Serum samples were collected 2 h after intraperitoneal LPS injection, and cytokine concentrations were measured. Data represent mean ± standard error (*n *= 3). There were no statistically significant differences in the cytokines tested. (C) Changes in ear thickness of individual mice after TPA treatment were determined on the indicated days. Data represent mean ± standard error (*n *= 8). (D) Neutrophils recruited to the peritoneal cavity following zymosan injection were recovered and counted. Data represent mean ± standard error (*n *= 3).

To assess the effects of myeloid-specific selenoprotein deficiency on *in vivo *pathology, control and *ΔTrsp*^*M *^mice were subjected to different models of the inflammatory response. The models employed in our study include LPS endotoxemia, chemical irritant (12-*O*-tetradecanoylphorbol-13-acetate [TPA]) dermatitis, and zymosan-induced peritonitis. In these experimentally induced inflammatory responses, *ΔTrsp*^*M *^mice manifested rates of mortality, and levels of cytokine production, local edema formation, and neutrophil infiltration that are comparable to those seen in control animals (Figure 4). Hence, despite selenoprotein deficiency and deregulated ROS generation in macrophages, *ΔTrsp*^*M *^mice appeared normal in macrophage-mediated inflammatory responses.

### Changes in gene expression in *ΔTrsp*^*M *^macrophages

**Figure 5:**
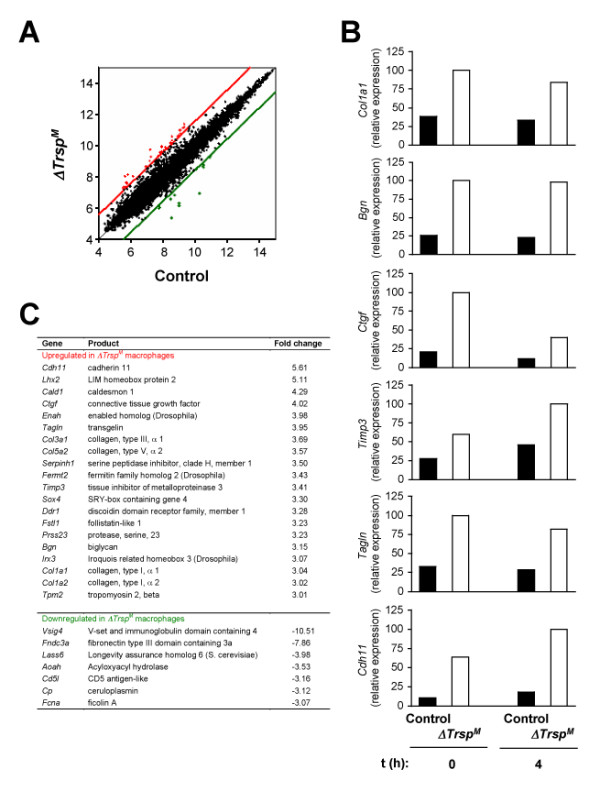
**Altered gene expression in *ΔTrsp*^*M *^macrophages**. (A) Genes upregulated (red spots) and downregulated (green spots) in *ΔTrsp*^*M *^BMDMs were shown in the scatter plots. Numbers on the ordinate and abscissa represent logarithmic values of the intensity of signal of individual microarray spots. The cut-off lines indicate the margins of gene expression ratio: 3-fold or higher (upregulated genes; red) and 3-fold or lower (downregulated genes; green). (B) The expression levels of genes identified in DNA microarray analysis were validated by real-time qPCR. Shown is the relative expression of genes in BMDMs left untreated and treated with LPS (100 ng/ml; 4 h). Data are representative of two experiments. All genes presented exhibited statistically significant differences in their expression levels in WT and *ΔTrsp*^*M *^BMDMs. (C) Genes and protein products whose expression is regulated by selenoproteins.

A recent study that used independently created *Trsp *mutant mouse lines also showed that Sec tRNA^[Ser]Sec ^ablation resulted in elevated ROS generation in macrophages and hepatocytes, yet its effects on cell survival and function were masked by compensatory induction of a cytoprotective transcription program mediated by NF-E2-related factor 2 [20]. The lack of discernable inflammatory phenotypes in *ΔTrsp*^*M *^mice may be attributable to such redundancy in cellular antioxidant mechanisms. We reasoned that, in addition to their known activities, selenoproteins expressed in macrophages provide other, nonredundant functions, the loss of which may be translated into gene expression patterns. To test this idea, we analyzed global gene expression profiles of control and *ΔTrsp*^*M *^macrophages. Mouse whole-genome DNA microarrays were used to determine mRNA levels in the two macrophage groups that were left unstimulated and stimulated with LPS. Specific sets of genes showed significantly higher or lower expression in *ΔTrsp*^*M *^macrophages (Figure 5A and data not shown). The data obtained from the DNA microarray experiments were verified by real-time qPCR analysis (Figure 5B).

The most salient feature of the genes showing aberrant expression in selenoprotein-deficient macrophages was that many were functionally related to the formation and remodeling of and cellular interaction with the extracellular matrix (ECM). In both resting and LPS-stimulated *ΔTrsp*^*M *^macrophages, these ECM-related genes were expressed at higher levels relative to control cells (Figure 5C), and included those that encode ECM components (collagen chains, extracellular proteoglycans, and secreted glycoproteins; *Col1a1*, *Col5a2*, *Bgn*, *Ctgf *and *Sparc*), inhibitors of ECM proteolysis (metalloendopeptidase and serine-type endopeptidase inhibitors; *Timp3*, and *Serpinh1*), and ECM-induced cytoskeletal remodeling (actin binding proteins; *Tagln*, *Enah*, and *Cald1*). A few other genes were detected as being expressed at lower levels in LPS-treated *ΔTrsp*^*M *^macrophages, but their repertoire did not point to any particular shared attributes (Figure 5C).

### Effects of selenoprotein deficiency on macrophage invasiveness

**Figure 6:**
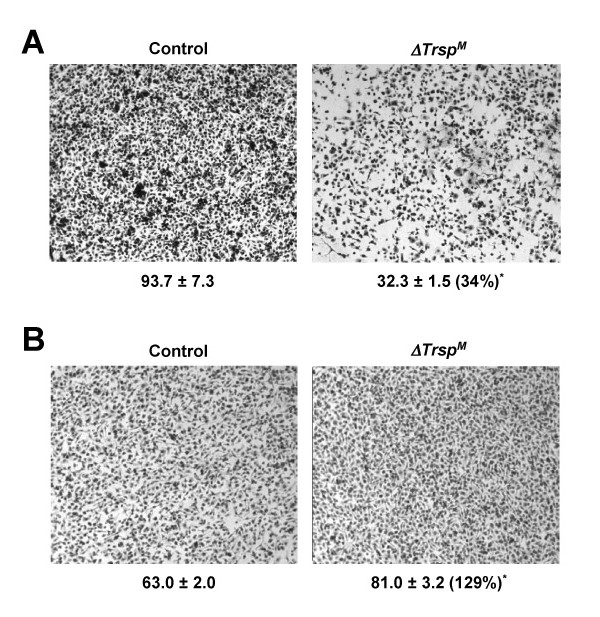
**Reduced invasion of *ΔTrsp*^*M *^macrophages in a protein gel matrix**. (A) Migration of BMDMs was analyzed by a transwell assay with a protein gel matrix (Matrigel) layer in the upper chamber. The number below each of the panels indicates relative macrophage migration and represents mean ± standard deviation (*n *= 3). *, *p *= 0.0003. The number in parenthesis indicates percentage of cell migration in the *ΔTrsp*^*M *^sample relative to the control sample. (B) Macrophage migration was analyzed by a transwell assay and the data presented as in *A*. but in the absence of a Matrigel layer. *, *p *= 0.0002.

The DNA microarray data obtained from the analysis of *ΔTrsp*^*M *^macrophages suggested that loss of selenoprotein expression alters macrophage-ECM interactions in such a way as to decrease matrix remodeling, and in favor of reinforcing the surrounding ECM. Those effects would also affect macrophage migration through the ECM and basement membrane. We tested this idea by comparing the invasiveness of control and *ΔTrsp*^*M *^macrophages in a protein gel matrix (Matrigel). Macrophages devoid of selenoprotein expression showed substantially reduced migration in gel-laden transwell chambers (Figure 6A). We also tested whether the gel invasion phenotype arised from a cell-intrinsic motility defect by repeating the assay without the gel in the transwell chamber; migration of *ΔTrsp*^*M *^macrophages in gel-free media was not reduced and rather slightly higher than that of control macrophages (Figure 6B). Thus, the changes of ECM-related gene expression in macrophages appear to impair the cells' migration properties only in an environment comprising ECM components.

## Discussion

The immune regulatory effects exerted by dietary selenium are complex, and appear to be determined by multiple variables associated not only with the micronutrient but also with the immune system. Despite the epidemiological link between selenium deficiency and inflammatory disorders, little insight has been gained as to the role of selenium and selenoproteins in the pathology, and the cell types wherein they serve protective and self-destructive functions. Inflammation is driven by multiple types of leukocytes and parenchymal cells of the immune system. We previously reported that T cells lacking Sec tRNA^[Ser]Sec ^and selenoprotein expression were defective in T cell receptor-induced proliferation, a key step for activating T cell-mediated immune responses [11]. In the current study, we determined the selenoproteins abundantly expressed in resting and activated macrophages, and identified a role for selenoproteins in macrophage gene regulation and invasive behavior.

Redox regulation is an inherent component of inflammatory signaling in macrophages [21]. In accord with the antioxidant activities of many selenoproteins, we observed elevated ROS generation in selenoprotein-deficient macrophages. However, this ROS dysregulation did not lead to overt phenotypes in the mouse models of inflammation that were used in this study. These results may imply that the levels of ROS and oxidative damage increased in *ΔTrsp*^*M *^macrophages are subthreshold for effects on inflammatory responses. Alternatively, there may exist fail-safe mechanisms in macrophages that act to maintain inflammatory events in the absence of selenoprotein function. It was indeed shown by others that cytoprotective enzymes that are induced in a manner dependent on the transcription factor NF-E2-related factor 2 can counter oxidative stress in Sec tRNA^[Ser]Sec^-deficient cells and thereby compensate for the loss of selenoproteins [20]. Whichever the case may be, selenoprotein function is likely dispensable for the onset as well as the termination of inflammatory responses, at least under the specific experimental settings employed in our tests. Of note, the selenoprotein deficiency of *ΔTrsp*^*M *^macrophages resulted in increased expression of ECM-related genes. Therefore, although the precise mechanism remains to be determined, selenoproteins serve nonredundant gene regulatory functions in macrophages.

Cell migration *in vivo *entails proteolytic remodeling of ECM. Matrix metalloproteinases (MMPs) and other groups of protein-degrading enzymes are well known for their role in cellular ECM invasion. Amongst the ECM-related genes overexpressed in *ΔTrsp*^*M *^macrophages are *Tagln *and *Timp3*, whose protein products were shown to inhibit the expression or activity of MMPs and thus suppress cell invasive activity [22,23]. In keeping with the high expression of these genes, *ΔTrsp*^*M *^macrophages displayed greatly diminished invasion in a protein gel matrix. Other ECM-related genes identified in our DNA microarray experiments are also likely to contribute to this phenotype. Invasive macrophages play a central pathogenic role in certain chronic inflammatory lesions: most notably foam cells in atherosclerotic plaques, and tumor-associated macrophages. Therefore, *ΔTrsp*^*M *^mice may show an altered severity or kinetics of disease in as-yet-unexplored experimental conditions, and serve as animal models of chronic human diseases that are associated with selenium deficiency.

## Conclusion

Selenium and selenoproteins may regulate immunity and tissue homeostasis through ECM-related gene expression and macrophage invasiveness.

## Methods

### Mice and primary macrophages

A C57BL/6 mouse line carrying floxed *Trsp *(*Trsp*^*fl*/*fl*^; designated as control) was described previously [24]. A transgenic C57BL/6 line carrying the *Lysozyme M-Cre *transgene [15] was from the Jackson Laboratory. These lines were mated to obtain *ΔTrsp*^*M *^mice. The maintenance and care of all mice were conducted under IACUC-approved protocols and in accordance with the National Institutes of Health institutional guidelines under the expert direction of Dr. John Dennis (NCI, NIH, Bethesda, MD, U.S.A.). BMDMs and PEMs were prepared and cultured as described [25]. The extent of *Trsp *deletion in each cell preparation were determined by qPCR analysis of the floxed region of the gene.

### ^75^Se-labeling and analysis of selenoproteins

Macrophages were incubated with 25 μCi/ml of ^75^Se for 24 h, lysed, the resulting protein extractions electrophoresed on gels, gels stained with Coomassie Blue R-250, vacuum dried and exposed to a PhosphorImager as described [14,26].

### Protein, mRNA, and ROS analysis

The level of IκBα, ERK, JNK, and p38 activation was determined by immunoblotting with antibodies specific to total and phosphorylated proteins (Cell Signaling Technology). Secreted proteins in culture media were assayed by SearchLight protein array analysis (Pierce). Total RNA was isolated using Trizol (Invitrogen). Microarray analysis was performed with GeneChip Mouse 430 2.0 Array (Affymetrix) at the Molecular Technology Microarray Laboratory (Frederick, MD). The data were normalized and statistically analyzed using software tools (Expression Console; Affymetrix) provided by the National Cancer Institute, Center for Cancer Research in collaboration with the National Institutes of Health, Center for Information Technology, Bioinformatics and Molecular Analysis Section. All microarray data discussed in this paper are available at the GEO repository at NCBI under the accession number GSE15610. Genes showing significantly different expression levels (*p *< 0.01) in WT versus *ΔTrsp*^*M *^macrophages in three independent hybridizations were chosen for validation by real-time qPCR, which was performed as described [25]. ROS were measured by flow cytometry and confocal microscopy using oxidation sensitive dye DCFDA as described [11].

### Inflammatory response in vivo

LPS endotoxemia was induced by intraperitoneal injection of *Escherichia coli *055:B5 LPS (50 mg/kg; Sigma) in phosphate-buffered saline (PBS). For acute edema formation, the right auricle was irritated by topical treatment with 2 μg of TPA in 20 μl of acetone. For control irritation, 20 μl of acetone was applied to the left auricle of the same animal. Ear thickness was measured using a dial thickness gauge (Mitutoyo). Changes in ear thickness were determined base on the following formula: Ear swelling on day X after TPA treatment = (thickness of the right ear on day X - thickness of the right ear on day 0) - (thickness of the left ear on day X - thickness of the left ear on day 0). To induce acute neutrophil infiltration, 0.5 mg of zymosan (Sigma) in 0.5 ml of PBS was injected intraperitoneally. Peritoneal exudate was recovered 4 h following injection and the number of neutrophils in the exudate was counted under bright-field microscopy.

### Cell invasion and migration assay

3 × 10^5 ^BMDMs in 200 μl of culture medium containing 0.5% fetal bovine serum were loaded onto the upper chamber of either a protein gel-coated BD BioCoat Matrigel Invasion Chamber (pore size, 8.0 μm; BD Biosciences) or a non-coated transwell plate (pore size, 8.0 μm; Corning). For invasion assay, 600 μl of culture medium containing 10% fetal bovine serum was placed in the lower well, and cells were incubated for 24 hr. The lower surface of the membrane was stained with 0.09% crystal violet and macrophages counted under bright-field microscopy. The numbers of macrophages in three independent image fields of the same size were counted to obtain the means and standard deviations.

### Statistical analysis

P-values between a pair of datasets were obtained from two-tailed Student's t-test using GraphPad Prism 4.0. Gene expression data are shown as the average ± standard deviation.

## Authors' contributions

BAC, MHY, YS, AS, JYK, RI, and JMP carried out the experiments. BAC, DLH, and JMP drafted the manuscript. BAC, VNG, DLH, and JMP designed the study and interpreted the data. All authors read and approved the final manuscript.

## References

[B1] Gordon S, Taylor PR (2005). Monocyte and macrophage heterogeneity. Nat Rev Immunol.

[B2] Hume DA (2006). The mononuclear phagocyte system. Curr Opin Immunol.

[B3] Mosser DM, Edwards JP (2008). Exploring the full spectrum of macrophage activation. Nat Rev Immunol.

[B4] Akira S, Uematsu S, Takeuchi O (2006). Pathogen recognition and innate immunity. Cell.

[B5] Creagh EM, O'Neill LA (2006). TLRs, NLRs and RLRs: a trinity of pathogen sensors that co-operate in innate immunity. Trends Immunol.

[B6] Hatfield DL, Berry ML, Gladyshev VN (2006). Selenium Its Molecular Biology and Role in Human Health.

[B7] Hoffmann PR, Berry MJ (2008). The influence of selenium on immune responses. Mol Nutr Food Res.

[B8] Hatfield DL, Carlson BA, Xu XM, Mix H, Gladyshev VN (2006). Selenocysteine incorporation machinery and the role of selenoproteins in development and health. Prog Nucleic Acid Res Mol Biol.

[B9] Xu XM, Carlson BA, Mix H, Zhang Y, Saira K, Glass RS, Berry MJ, Gladyshev VN, Hatfield DL (2006). Biosynthesis of selenocysteine on its tRNA in eukaryotes. PLoS Biol.

[B10] Carlson BA, Novoselov SV, Kumaraswamy E, Lee BJ, Anver MR, Gladyshev VN, Hatfield DL (2004). Specific excision of the selenocysteine tRNA[^[Ser]Sec ^(*Trsp*) gene in mouse liver demonstrates an essential role of selenoproteins in liver function. J Biol Chem.

[B11] Shrimali RK, Irons RD, Carlson BA, Sano Y, Gladyshev VN, Park JM, Hatfield DL (2008). Selenoproteins mediate T cell immunity through an antioxidant mechanism. J Biol Chem.

[B12] Kryukov GV, Castellano S, Novoselov SV, Lobanov AV, Zehtab O, Guigó R, Gladyshev VN (2003). Characterization of mammalian selenoproteomes. Science.

[B13] Hoffmann PR, Höge SC, Li PA, Hoffmann FW, Hashimoto AC, Berry MJ (2007). The selenoproteome exhibits widely varying, tissue-specific dependence on selenoprotein P for selenium supply. Nucleic Acids Res.

[B14] Gladyshev VN, Stadtman TC, Hatfield DL, Jeang KT (1999). Levels of major selenoproteins in T cells decrease during HIV infection and low molecular mass selenium compounds increase. Proc Natl Acad Sci USA.

[B15] Clausen BE, Burkhardt C, Reith W, Renkawitz R, Förster I (1999). Conditional gene targeting in macrophages and granulocytes using LysMcre mice. Transgenic Res.

[B16] Hsu HY, Wen MH (2002). Lipopolysaccharide-mediated reactive oxygen species and signal transduction in the regulation of interleukin-1 gene expression. J Biol Chem.

[B17] Park HS, Jung HY, Park EY, Kim J, Lee WJ, Bae YS (2004). Direct interaction of TLR4 with NAD(P)H oxidase 4 isozyme is essential for lipopolysaccharide-induced production of reactive oxygen species and activation of NF-κB. J Immunol.

[B18] Brigelius-Flohé R, Friedrichs B, Maurer S, Schultz M, Streicher R (1997). Interleukin-1-induced nuclear factor kappa B activation is inhibited by overexpression of phospholipid hydroperoxide glutathione peroxidase in a human endothelial cell line. Biochem J.

[B19] Esworthy RS, Aranda R, Martín MG, Doroshow JH, Binder SW, Chu FF (2001). Mice with combined disruption of Gpx1 and Gpx2 genes have colitis. Am J Physiol Gastrointest Liver Physiol.

[B20] Suzuki T, Kelly VP, Motohashi H, Nakajima O, Takahashi S, Nishimura S, Yamamoto M (2008). Deletion of the selenocysteine tRNA gene in macrophages and liver results in compensatory gene induction of cytoprotective enzymes by Nrf2. J Biol Chem.

[B21] Janssen-Heininger YM, Mossman BT, Heintz NH, Forman HJ, Kalyanaraman B, Finkel T, Stamler JS, Rhee SG, Vliet A van der (2008). Redox-based regulation of signal transduction: principles, pitfalls, and promises. Free Radic Biol Med.

[B22] Nair RR, Solway J, Boyd DD (2006). Expression cloning identifies transgelin (SM22) as a novel repressor of 92-kDa type IV collagenase (MMP-9) expression. J Biol Chem.

[B23] Johnson JL, Sala-Newby GB, Ismail Y, Aguilera CM, Newby AC (2008). Low tissue inhibitor of metalloproteinases 3 and high matrix metalloproteinase 14 levels defines a subpopulation of highly invasive foam-cell macrophages. Arterioscler Thromb Vasc Biol.

[B24] Kumaraswamy E, Carlson BA, Morgan F, Miyoshi K, Robinson GW, Su D, Wang S, Southon E, Tessarollo L, Lee BJ, Gladyshev VN, Hennighausen L, Hatfield DL (2003). Selective removal of the selenocysteine tRNA[^[Ser]Sec ^gene (*Trsp*) in mouse mammary epithelium. Mol Cell Biol.

[B25] Park JM, Greten FR, Li ZW, Karin M (2002). Macrophage apoptosis by anthrax lethal factor through p38 MAP kinase inhibition. Science.

[B26] Moustafa ME, Carlson BA, El-Saadani MA, Kryukov GV, Sun QA, Harney JW, Hill KE, Combs GF, Feigenbaum L, Mansur DB, Burk RF, Berry MJ, Diamond AM, Lee BJ, Gladyshev VN, Hatfield DL (2001). Selective inhibition of selenocysteine tRNA maturation and selenoprotein synthesis in transgenic mice expressing isopentenyladenosine-deficient selenocysteine tRNA. Mol Cell Biol.

